# Composite HPMC-Gelatin Films Loaded with Cameroonian and Manuka Honeys Show Antibacterial and Functional Wound Dressing Properties

**DOI:** 10.3390/gels11070557

**Published:** 2025-07-19

**Authors:** Joshua Boateng, Sana Khan

**Affiliations:** School of Science, Faculty of Engineering and Science, University of Greenwich, Medway, Kent ME4 4TB, UK

**Keywords:** antibacterial, Cameroon honey, composite gel, film dressing, Manuka honey, gelatin, HPMC, wound healing

## Abstract

Antimicrobial resistance in infected chronic wounds present significant risk of complications (e.g., amputations, fatalities). This research aimed to formulate honey-loaded hydrocolloid film comprising gelatin and HPMC, for potential treatment of infected chronic wounds. Honeys from different sources (Cameroonian and Manuka) were used as the bioactive ingredients and their functional characteristics evaluated and compared. The formulated solvent cast films were functionally characterized for tensile, mucoadhesion and moisture handling properties. The morphology and physical characteristics of the films were also analyzed using FTIR, X-ray diffraction and scanning electron microscopy. Antibacterial susceptibility testing was performed to study the inhibition of *Escherichia coli*, *Pseudomonas aeruginosa*, and *Staphylococcus aureus* by honey components released from the films. The % elongation values (8.42–40.47%) increased, elastic modulus (30.74–0.62 Nmm) decreased, the stickiness (mucoadhesion) (0.9–1.9 N) increased, equilibrium water content (32.9–72.0%) and water vapor transmission rate (900–298 gm^2^ day^−1^) generally decreased, while zones of inhibition (2.4–6.5 mm) increased with increasing honey concentration for 1 and 5% *w*/*v*, respectively. The results generally showed similar performance for the different honeys and demonstrate the efficacy of honey-loaded hydrocolloid films as potential wound dressing against bacterial growth and potential treatment of infected chronic wounds.

## 1. Introduction

Chronic wounds impose a significant health burden on healthcare systems, as the population ages coupled with increase in underlying pathologies such as Type 2 diabetes. Furthermore, chronic wounds can be exacerbated by persistent infection which stalls the natural progression of wound healing [1,2]. The problem of antimicrobial resistance (AMR) (including infected chronic wounds) is of current clinical and public health concern which can result in complications such as amputations and fatalities. The WHO now counts AMR among the top 10 global diseases requiring urgent attention [3]. Honey has been used in medicine for centuries and has been proposed for treating chronic wounds, due to its strong antibacterial and wound healing properties and provides a relatively cheaper alternative to antibiotics given the current lack of new antibiotics able to overcome AMR coming through the drug development pipeline. Research in recent years has concluded that honey is not only a natural food material but that it also functions as a biological wound dressing with several bioactive components that can expedite the healing process [4,5] with honey-based dressings commercially currently available on the market. Such effects include accelerated healing [6], infection clearance [7], anti-inflammatory [8] and debriding performance on wounds [9]. The major biological and therapeutic properties of honey are summarized in Table S1.

There is now renewed interest in using dressings loaded with honey for different types of honey for potential wound healing purposes [10]. Chopra et al., [11] developed chitosan/polyvinyl alcohol (PVA)-based films loaded with honey for wound healing application. The honey loaded films showed acceptable properties including water vapor transmission rate, tensile properties and smooth surface morphology and demonstrated activity against *Staphylococcus aureus*. Lotfinia and co-workers [12] developed an alginate-based hydrogel loaded with honey and anthocyanin to monitor and treat bacterial infections in wounds. The resulting hydrogel exhibited pH responsiveness, antioxidant, antibacterial properties and demonstrated appropriate biocompatibility and therefore potential for treating infected chronic wounds. Other reported studies of honey-based dressings include composite PVA–sucrose–honey dressing [13]; honey/PVA hybrid wound dressings that also exhibited controlled release of antibiotics [14]; composite dressing comprising PVA and crosslinked chitosan loaded with honey and allantoin [15]. In addition, Lo and co-workers developed patches containing Kelulut honey using cellulose and poly(lactic-co-glycolic acid as film forming polymers [16].

All the above studies involved the use of honey from single sources and to the best of our knowledge, no study has reported composite film dressings comprising HPMC and gelatin, loaded with two types of Cameroonian honeys and comparing with Manuka honey. In an interesting study, Kolour and co-workers [17] designed bilayered multifunctional film dressings comprising electrospun gelatin and polycaprolactone nanofibers reinforcing enzyme crosslinked gelatin hydrogel layer containing curcumin and honey. In this case, the honey was added as a lubricant (rather than as antimicrobial) to prevent adhesion to the tissue, thus enabling easy dressing change without damage to sensitive newly formed tissues. However, no antibacterial action of the honey-loaded dressings was investigated in their study, unlike in this study where the dressings were tested against three common wound infection causative bacteria. In another study, dos Santos and co, [18] prepared cryogels comprising PVA combined with either sodium carboxymethylcellulose or gelatin, loaded with Brazilian honey and characterized using Fourier transform infrared spectroscopy (FTIR), differential scanning calorimetry, swelling and antimicrobial action against a single bacteria (*S. aureus*) compared to single polymer cryogels. They showed that only the composite PVA cryogels loaded with honey showed antimicrobial action against the model infection causing bacteria.

The most common honey-based dressing products currently on the market are Manuka and Surgi-honey, which possess well defined bioactive properties and are effective for treating wounds across Australia and Europe [19,20]. However, no other commercial dressings containing honeys from other geographical regions are available. In a previous study [21], we investigated the functional physical and antibacterial properties of two types of Cameroonian honeys and compared with those of the standard commercially available Manuka honey. The results from this study demonstrated that the Cameroonian honeys, which were significantly cheaper than the Manuka honey, exhibited antibacterial physico-chemical properties similar to the commercially available Manuka honey, confirming their potential as an effective alternative to Manuka honey especially in resource limited parts of the world such as sub-Saharan Africa. The aim of this study was to develop a potentially effective but affordable treatment of infected wounds caused by antibiotic resistant bacteria, which is a leading cause of amputations and deaths in many African countries [21]. This involved the formulation, optimization, and characterization of Cameroonian honey-based composite hydrocolloid films comprising HPMC and gelatin and comparing with Manuka honey, which is commercially available. To the best of our knowledge, this is the first study comparing composite HPMC-gelatin based hydrocolloid film dressings loaded with two types of honey from Cameroon and comparing with Manuka loaded equivalent films.

## 2. Results and Discussion

### 2.1. Visual Evaluation of Films

**Figure 1:**
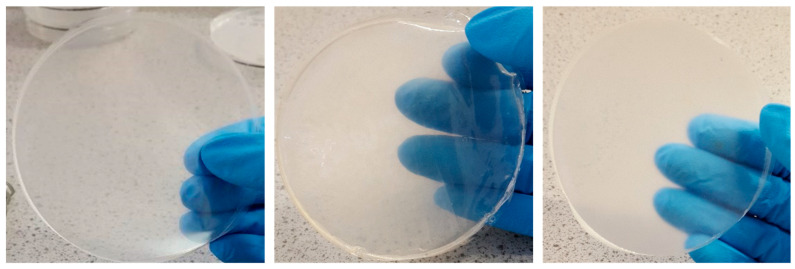
Visual images of selected representative honey-loaded films (prepared from 1% *w*/*v* gels comprising HPMC: gelatin 3:1—see Table 1), containing the three types of honey (CWH4, CSH8 and MH10, respectively—see Table 2) used in the study. The images showed good compatibility between the honey and the polymers and demonstrated that the films were moderately transparent [22].

Various visual investigations were conducted to evaluate the homogeneity, flexibility, and softness of the films, and Figure S1 showed that the blank films prepared from 1% *w*/*v* gels showed appropriate characteristics than the films obtained from 2% *w*/*v* gels. BLA1 was clear, brittle, homogenously dispersed and retained shape, BLA2 was clear, flexible, homogenously dispersed and retained shape, while BLA3 was clear, slightly brittle and homogenously dispersed). Figure S2 shows preliminary HPMC only films loaded with honey which did not yield ideal physical properties; therefore, only composite HPMC: gelatin films were used for loading the honey. Subsequently, composite films prepared from 1 and 2% *w*/*v* gels containing different ratios of HPMC: gelatin, loaded with 5% honey were investigated to identify the optimum film formulation for further studies. The results (Figure S3) showed that only the 3:1 HPMC: gelatin films prepared from 1 % *w*/*v* gels showed ideal properties suitable for easy handling and application and chosen as optimum formulation for further study. Subsequently, honey-excipient compatibility studies were undertaken with the optimum 1% 3:1 HPMC: gelatin films by adding different concentrations of the three types of honey with representative digital images shown in Figure 1.

Though the 1:1 ratio of HPMC and gelatin also resulted in an ideal blank film, the addition of honey did not produce adequate results due to the highly viscous gel formed between honey and gelatin which made it difficult to pour and also did not yield uniform films. Generally, all solutions of 3:1 HPMC: gelatin produced homogenously dispersed gels with little to no air bubbles. As expected, the viscosity of the gels increased with increasing honey content. In addition, films appeared more flexible and durable with increasing honey content, attributed to its plasticizing action; however, there was some loss in transparency with increasing honey concentration (Figure 1).

### 2.2. Physical and Mechanical Characterization of Hydrocolloid Films

#### 2.2.1. Film Thickness and Folding Endurance

**Figure 2:**
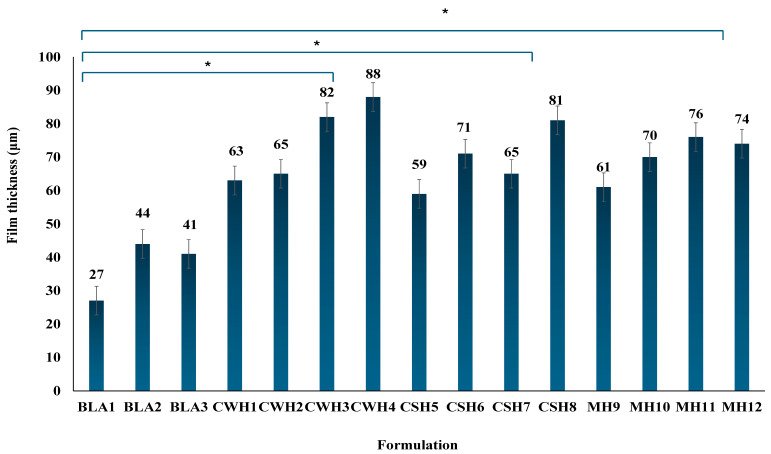
Mean film thickness of final optimized formulations (*n* = 3; ±SD) showing differences between the blank and honey loaded films. Generally, addition of honey resulted in significant increases in thickness compared to the blank films (* *p* < 0.05 showing significant difference between the honey-loaded film with highest concentration of honey to the blank films).

The thickness of the final formulations was measured in five locations to confirm their uniformity and consistency, and the mean results are shown in Figure 2. This is critical as thickness affects various functional properties including mechanical strength, ability to adhere to and achieve proper fit to the wound, swelling and exudate handling behavior and consistent drug release [23,24]. All the blank films showed significantly (*p* < 0.05) lower thickness values compared to the honey-loaded films. All the honey-loaded films exhibited uniform and consistent thickness, with increased honey content resulting in higher thickness values; however, the increase between consecutive concentrations (e.g., CW1/CW2, CS5/CS6, MH9/MH10) were not significant ((*p* > 0.05), whilst the difference between the lowest and highest honey concentrations (e.g., CW1 and CW4) were significant (*p* < 0.05) for all three honey types.

The folding endurance of a hydrocolloid film is a quantitative measurement that indicates the flexibility of a film. This value was determined by repeatedly folding each film in the same position of a total number of 300 times. Each film exhibited a folding endurance >300, thus indicating suitable flexibility and strength. Amruth et al. [25] reported that folding endurance values >300 were deemed appropriate for easy handling and ensured durability and subsequent application to the wound. Furthermore, the honey present in the films exhibited similar properties to a plasticizer, where an increase in honey concentration resulted in a more flexible film. Therefore, it can be concluded that optimized honey-loaded hydrocolloid films demonstrated acceptable folding endurance requirements for wound dressings and are acceptable systems for wound application without sticking together breaking prior to being applied.

#### 2.2.2. Mechanical Properties

**Figure 3:**
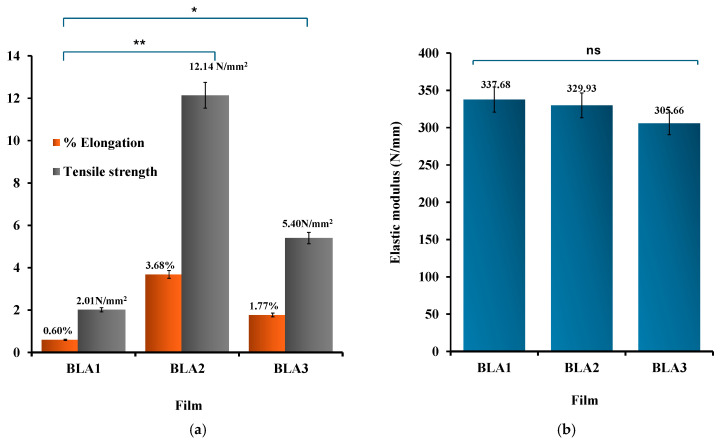
(**a**) Elongation at break, tensile strength and (**b**) elastic modulus (right) for blank films (*n* = 3; ±SD). The differences in tensile strength/% elongation when comparing between BLA1 and BLA2/BLA3 were significant at *p* < 0.05 (*) and *p* < 0.01 (**), respectively, while no significant (ns) differences (*p* > 0.05) were observed between the elastic modulus values for the three blank films.

Mechanical properties were characterized using a texture analyzer to determine the % elongation at break, tensile strength, and elastic modulus of the films. Ideal mechanical properties for wound dressings include durability, flexibility, elastic and stress resistance to cope with external forces efficiently [26]. In addition, wound dressings that possess such properties ensure the wound site is well protected and enable the patient and/or carer to handle the dressing effectively with little damage to the dressing prior to application as well as reduces chances of damage to newly formed skin tissue [27,28]. Figure 3 displays the tensile profiles (% elongation at break, tensile strength, and elastic modulus) for the blank films. The results for % elongation and tensile strength for the blank films did not follow a logical pattern in terms of increasing HPMC concentration especially between BLA1 and BLA3. It is possible that there is an optimal combination between HPMC and gelatin beyond which the concentration of the main film forming polymer (HPMC) did not have a significant impact on the mechanical properties; however, this will require future optimization using design of experiment statistical tools, to help determine the optimum formulation design space using the polymer ratios as independent (input) variable and the mechanical properties as the dependent (output) variables. Further, it will be useful to compare the composite films with gelatin only films to evaluate the direct contribution of gelatin to the mechanical properties.

The elastic modulus values were all greater than 300 Nmm, suggesting very strong and rigid films [29]; however, all values were the same and within the measurement error for BLA1, BLA2, and BLA3 and therefore not used in selecting the optimum blank formulation for honey loading and further investigation. The % elongation results showed interesting profiles, with BLA2 exhibiting significantly (*p* < 0.05) higher % elongation at break compared to BLA1 and BL3, indicating more flexibility and reduced brittleness. Similarly, BLA2 also showed significantly (*p* < 0.05) higher tensile strength than BLA1 and BLA3. The tensile strength is used to measure the film’s ability to tolerate rupture and is a measure of the force required to tear the film apart [30]. Based on BLA2 exhibiting the highest tensile strength and % elongation, it was further investigated with different concentrations of honey.

**Figure 4:**
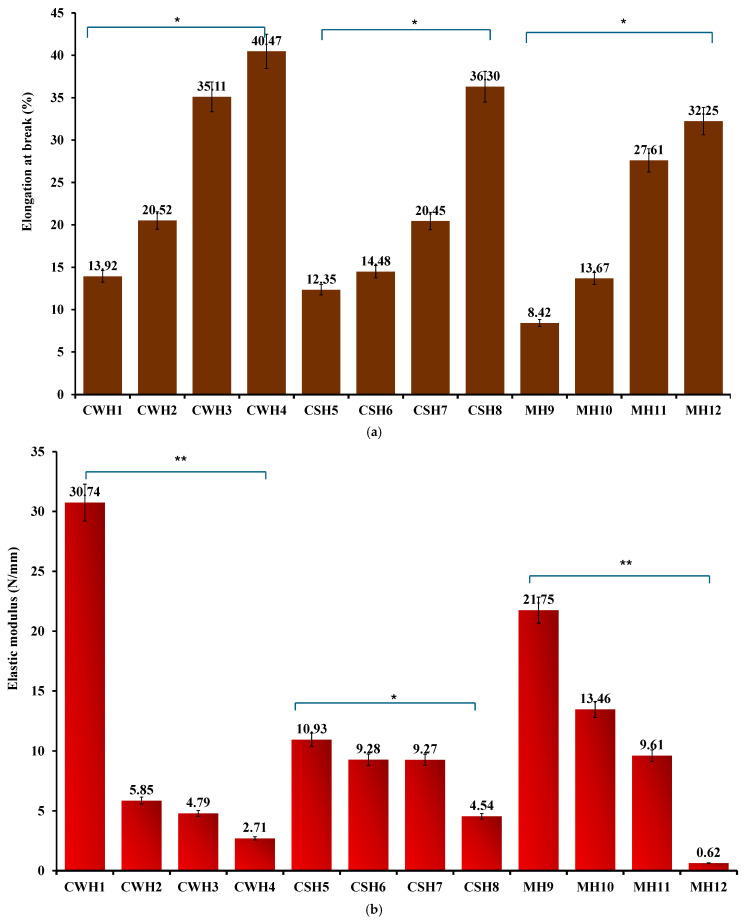

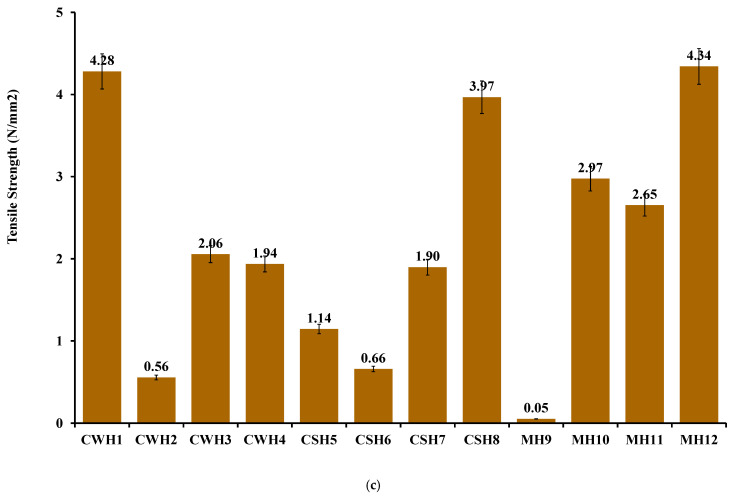
(**a**) % Elongation at break (**b**) elastic modulus; (**c**) tensile strength of honey-loaded hydrocolloid films. Significant difference between the films with lowest and highest concentrations of honey at *p* < 0.05 (*) and *p* < 0.01 (**), respectively.

The addition of honey caused significant changes to the mechanical properties of the films. Honey, a viscous and complex liquid, functioned as a plasticizer to the films by increasing their flexibility, durability, and thickness and overall toughness. Figure 4a–c shows the tensile profiles for the final honey-loaded hydrocolloid films. The presence of honey significantly (*p* < 0.05) increased the % elongation at break values of the films compared to the blank films. Further, increasing honey concentrations resulted in significant increase in percent elongation for all three honey types, while the elastic modulus (Figure 4b) significantly decreased with increasing honey concentrations. The significant difference was particularly evident between the lowest and highest concentrations of honey. This is further evidence that honey also functions indirectly as a plasticizer due to its hygroscopic effect that enables it to retain more water [31] improving the flexibility of the films together with gelatin, a polymer commonly used as a gelling agent [26]. Plasticizers function by interfering with the intra polymer chain interactions, increasing specific volume, decreasing glass transition temperature and therefore increasing polymer chain mobility with corresponding increase in flexibility (% elongation) and decrease in elastic modulus [32]. It was anticipated that the tensile strength should decrease with an increase in honey concentration; however, unlike the % elongation and elastic modulus, no definite trends were observed between the honey concentrations and tensile strength (Figure 4c). However, the elastic modulus, which gives some indication of strength and rigidity showed a logical trend to help evaluate the effects of increasing honey concentrations. That notwithstanding, all the honey-loaded films showed lower tensile strength values compared to the blank films, which confirms the plasticizing action of honey on the hydrocolloid films.

Based on the tensile profiles, the hydrocolloid films can be used for covering minor wounds as they can endure moderate frictional stress (highest percentage elongation shown at 30–40% in CWH4, CSH8, and MH12) such as daily wear and can withstand external forces without breaking. This will ensure the wound site is protected against further pain and damage [27,28].

### 2.3. In Vitro Wound Adhesion

**Figure 5:**
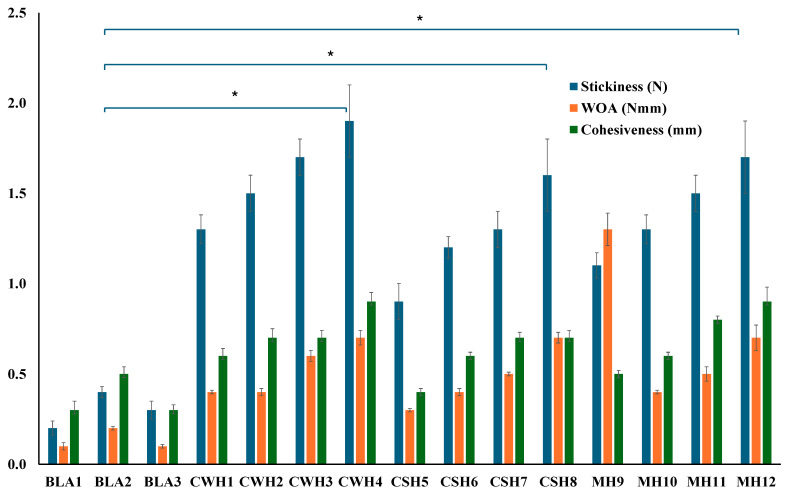
Mucoadhesion of hydrocolloid films with SWF. Significant difference between the blank films and films with the highest concentrations of honey at *p* < 0.05 (*).

The adhesive behavior of dressings is an important property to ensure prolonged residence time on the wound surface to allow enough time to exert its therapeutic action, which reduces the need for too frequent dressing changes. On the other hand, very high adhesion can result in pain during dressing change as well as potential damage to newly formed sensitive skin tissue [29,30]. Therefore, an appropriate balance is required in the adhesion performance of dressings when applied. Literature suggests that strong bonds are essential for successful mucoadhesion, such as the use of polymers containing H-bonding sites, efficient chain flexibility, anionic charges, and polymers that have high spreadability over mucosal surfaces [27,28]. Mucoadhesion is primarily reliant on the extent of diffusion and interfacial thickness of the two surfaces. The gelatin gel surface was equilibrated with SWF to almost mimic wound exudate. Figure 5 displays the work of adhesion (WOA) representing the area the curve, stickiness representing the peak adhesive force (PAF), and cohesiveness (travel distance) profiles for the blank and various honey loaded films.

Among the three blank films, BLA2 showed the highest WOA and cohesiveness. This could be attributed to BLA2 having the highest amount the hydrophilic HPMC, a well-known adhesive polymer. Furthermore, BLA2 also had the highest thickness values and was the strongest blank film with the highest tensile strength and % elongation values, therefore likely formed stronger hydrogen bonds with the simulated wound surface. All the honey-loaded films showed significantly higher adhesive profiles compared to the three blank films (BLA1, BLA2, BLA3). Figure 5 further reveals some interesting trends for honey-loaded films, with adhesion generally increasing as honey concentration increased for all three types of honey. This can be attributed to the ability of honey to increase the hydration and swelling behavior of the films, owing to honey’s humectant properties allowing easy interpenetration of the polymer chains of the films with the model moist wound surface and eventual formation of hydrogen bonds due to the availability of many OH groups.

### 2.4. Moisture Handling Properties

Modern moist dressings should have the ability to absorb exudate from the wound, retain enough within the hydrated and swollen matrix to maintain a moist wound environment essential for wound healing progression. This is indicated by measuring the equilibrium water content (EWC) which is the dressing’s water holding capacity after 24 h [2]. However, the dressing should also be able to transmit enough moisture into the atmosphere and this ability of the dressing to transmit moisture from the wound into the surrounding atmosphere is described the water vapor transmission rate (WVTR) [33]. Low WVTR values could result in the collection of excessive amounts of exudate which could cause maceration of healthy surrounding skin and also prone to infection. On the contrary, extremely high WVTR values could result in the wound bed drying out caused by excessive loss of fluid from the swollen dressings’ matrix. Table 3 demonstrates the EWC and WVTR for the different honey-loaded formulations. It can be deduced from the table that an increase in EWC is seen with an increase in honey concentration, which is attributed to the humectant property of honey owing to its hydrophilic components, mainly the presence of sugars. Boateng et al. [34], showed that moisture content generally increased with increasing concentrations of plasticizer (glycerol) which also has known humectant properties. Therefore, the highly hydrophilic nature of the honey components allowed the films to retain more moisture as the honey concentration increased.

For the WVTR, the values ranged between 320 and 900 gm^2^ day^−1^. The results also show that the honey–loaded films generally showed higher values than the blank films, except for MH11 which showed lower value of 298 ± 37 gm^2^ day^−1^ compared to 320 ± 60 gm^2^ day^−1^ for the optimized blank films (BLA2). Generally, the WVTR values were below the recommended rate of 2000–2500 g/m^2^ day^−1^ for highly exuding chronic wounds and therefore will be more suitable for moderately to low exuding wounds. In the case of infected highly exuding wounds, the honey-loaded films could be applied over a short period of time (maximum of 24 h), covered with a secondary dressing to tackle any infection. The film dressing can subsequently be changed to a more absorbent dressing such as wafer or foam, to avoid excess collection of exudate underneath the dressing, while still maintaining a moist wound environment [35].

### 2.5. Analytical Characterization

#### 2.5.1. Scanning Electron Microscopy (SEM)

**Figure 6:**
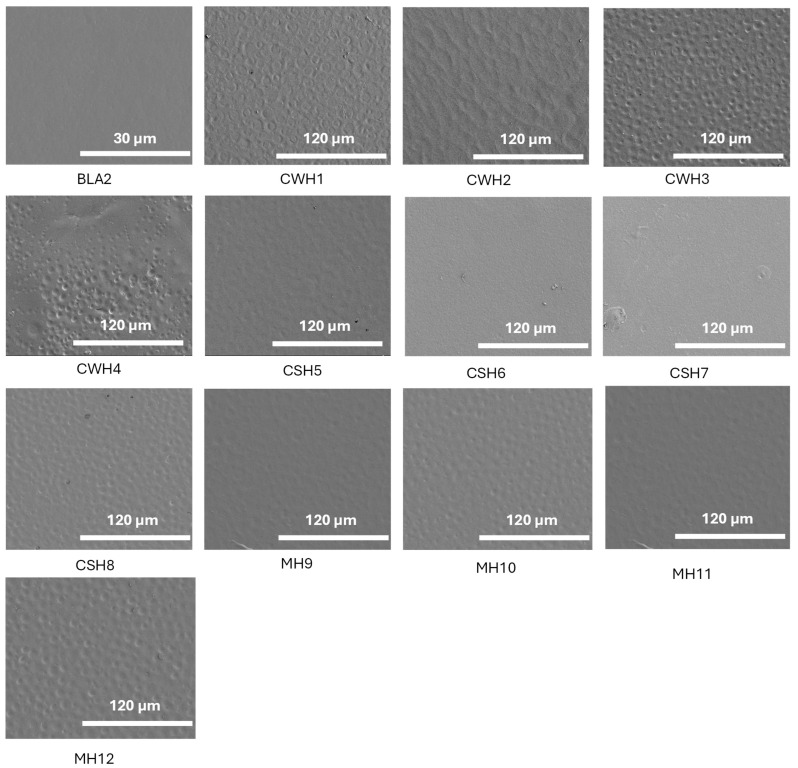
SEM images showing surface morphology of the blank and honey-loaded films. The figure demonstrates smooth homogeneous appearance for the blank film while the honey-loaded films showed circular patches on the surfaces but no obvious pores. The figure clearly shows how water evaporates during the sample preparation process, which is normal for the solvent casting approach for preparing films. Overall, both the blank and honey-loaded films did not exhibit any major deformities.

SEM images for hydrocolloid films (1% *w*/*v*) are shown in Figure 6. Initially, blank films were scanned to compare the surface morphology at different magnifications until a high-resolution image was obtained. The blank film was examined at ×1.5 k, whereas the honey-loaded films were evaluated at ×400 k. The resulting images give an indication of the magnitude and form of the samples at a nanometer scale and reveal in-depth information about the surface topography. By considering the topography of the selected optimized blank film (BLA2), a uniform and smooth appearance can be observed. This suggests that the combination of HPMC and gelatin in the composite gel blended efficiently resulting in homogeneous film surface. It was observed that almost all honey-loaded formulations produced spherical impressions; however, these were uniform throughout the film surface and showed no pores. This suggests that the addition of honey has consequently affected the film at the molecular level which was expected to affect other functional performance of the films including hydration and swelling, adhesion, EWC and WVTR. No significant difference can be seen between the different honey types; however, the spherical impressions on the CWH films were more prominent. Overall, the SEM images confirmed the visual observations made earlier from the digital images captured using a camera suggesting successful composite gel formulation and subsequent drying to obtain the uniform films.

#### 2.5.2. Fourier Transform Infrared Spectroscopy (FTIR)

**Figure 7:**
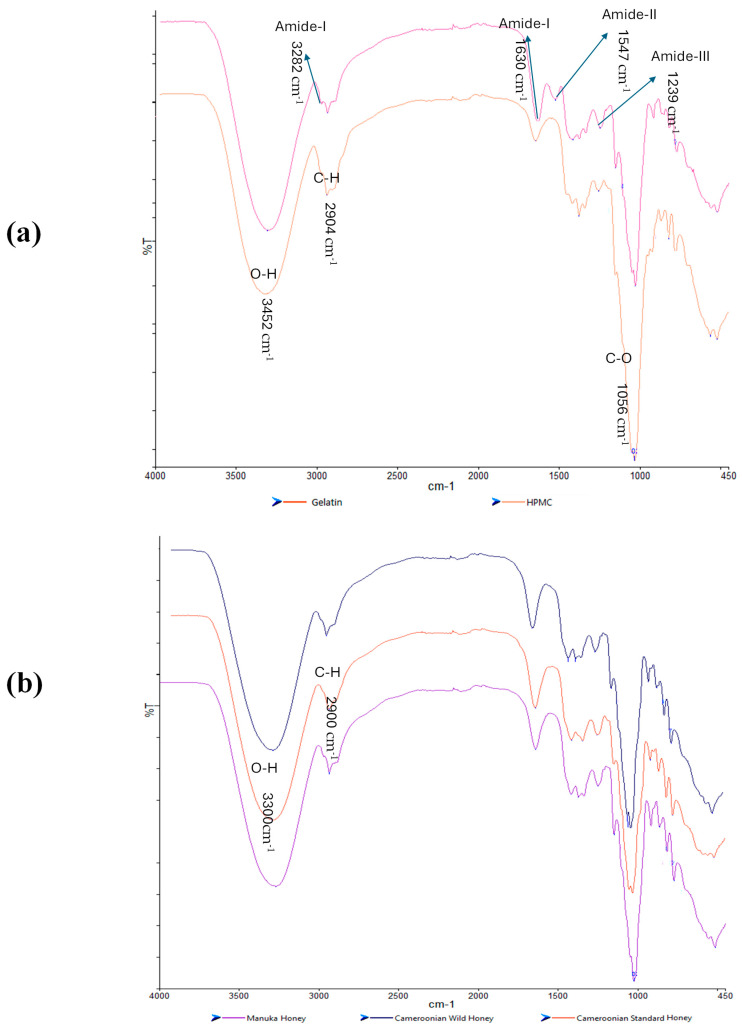
FTIR spectra of the pure starting materials (**a**) gelatin and HPMC; (**b**) three honey types.

FTIR spectroscopy was used to analyze and evaluate potential molecular interactions between the different film components by considering the band shifts in a specific functional group to help confirm their compatibility. The FTIR spectra for the starting materials (HPMC, gelatin and pure honey) are shown in Figure 7a,b. HPMC exhibited peaks for C-O stretching at 1056 cm^−1^, C-H stretching at 2904 cm^−1^, and O-H stretching at 3452 cm^−1^ while gelatin showed an amide-A band at a wavelength 3282 cm^−1^, another amide-I at a wavelength of 1630 cm^−1^, amide-II at a wavelength of 1547 cm^−1^, and an amide-III at a wavelength of 1239 cm^−1^. The three honey types showed similar spectra with no distinct peaks unique for any of the honey types, which could be attributed to the major components in honey being sugars with the other constituents present in trace quantities not detectable by FTIR.

**Figure 8:**
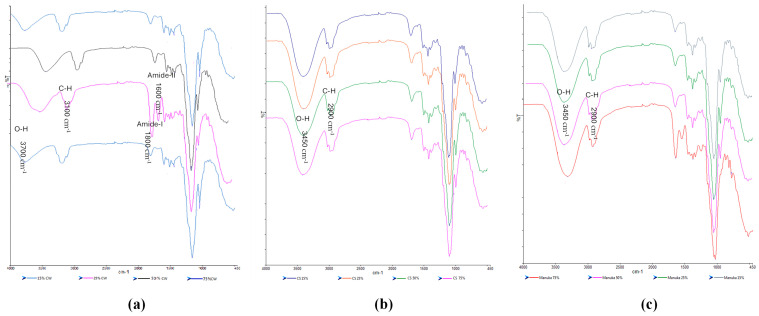
FTIR spectra of the honey-loaded films (**a**) CW, (**b**) CS, and (**c**) MH containing different concentrations of the honeys.

For the honey-loaded films (Figure 8a–c), unique peaks present in the honey but not in the HPMC/gelatin spectra can be observed; for example, the peak closest to 1000 cm^−1^. The peak at 3282 cm^−1^ for pure gelatin showed a chemical shift around a wavelength of 3100 cm^−1^ in the honey-loaded films suggesting possible interaction due to the hydrogen bonds between the OH groups present in both honey and gelatin. The CW loaded films in particular showed more significant shifts to higher wavenumbers for the O-H and C-H peaks (especially for the CW1 formulations), while the CS and MH loaded films were not too different from the starting materials. Overall, the FTIR spectra showed that the honey and the starting polymer were generally compatible, confirming the homogeneous nature of the films as observed from the digital and SEM images.

#### 2.5.3. XRD Results

**Figure 9:**
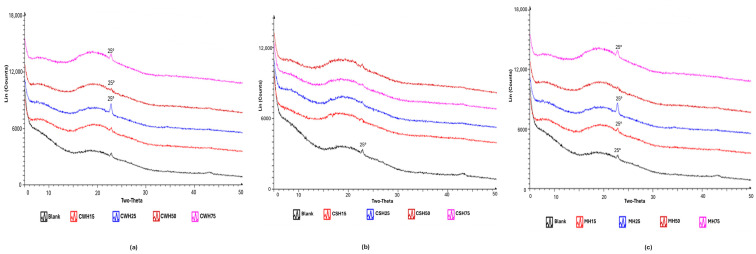
XRD diffractograms of honey-loaded films (**a**) CW, (**b**) CS and (**c**) MH containing different concentrations of the honeys.

XRD was used to determine the physical form (amorphous or crystalline nature) of the blank and honey-loaded films. The diffractograms (Figure 9a–c) showed broad halo peaks for all films confirming the amorphous characteristics of all formulations. This is to be expected as both HPMC and gelatin are known amorphous polymers, and the honey present is expected to be molecularly dispersed within the hydrophilic polymer matrix and therefore not expected to exhibit any crystalline peaks. Amorphous forms for polymeric formulations for use as wound dressings present several advantages including ease of hydration and swelling, mucoadhesion, moisture handling and release of loaded active ingredients. On the other hand, the amorphous form is less stable and may revert back to the more stable crystalline form, therefore storage conditions need to be properly controlled. 

### 2.6. Antibacterial Studies

The main objective of this study was to confirm that honey-loaded films will show similar antibacterial action against common wound infection bacteria, to the pure honeys as shown in previous study [21]. Table 4 shows the results of the zone of inhibition (ZOI) studies for the films comparing the BLA films with the honey-loaded films against *E. coli*, *P. aeruginosa*, and *S. aureus* while representative images depicting the ZOIs of the honey-loaded films against the 3 infection causation organisms are shown in Figures S4–S6.

The ZOIs were similar to those of the pure honey samples as previously reported [21]. This shows that the composite HPMC: gelatin films were able to release the antibacterial components present in honey to exert antimicrobial action and confirmed that the gel and film formulation steps did not alter the functional performance of the three honey samples. This confirms our hypothesis that Cameroonian and Manuka honey-loaded hydrocolloid dressings have potential as antimicrobial dressings to treat infected chronic wounds. However, the release profiles of one or more of the known bioactive components from the composite films, how long the film can remain active following application on a wound and how this could influence the frequency of dressing dressings, will need to be investigated in a future study. Comparison of the ZOIs of the honey-loaded films to that of the pure honey samples previously reported showed that the ZOIs of the films against the respective bacteria, were significantly lower (*p* < 0.01) than that of the pure honeys. This will suggest that the films may have a controlled release effect following hydration and swelling on the agar plate. To further characterize the antimicrobial action, the MIC of the different honey-loaded films was evaluated using the Kirby and Bauer standardized method for bacteria (*E. coli*, *P. aeruginosa*, and *S. aureus*). MIC is critical in bacterial susceptibility as it determines the concentration required to terminate bacterial chemical processes in the lowest possible amount. However, Figure S7 shows that none of the film samples tested demonstrated an effective MIC as all the test tubes remained cloudy. This indicates that the honey-loaded film strips cut out for the tests did not contain enough antibacterial agents from honey at the MIC to produce an inhibitory effect. Therefore, further studies using whole films rather than small strips and diluting them down to relevant levels will be required.

## 3. Conclusions

This research aimed to formulate an ideal honey-loaded bioactive hydrocolloid film dressings obtained from composite polymer gels comprising both gelatin and HPMC, for potential treatment of infected chronic wounds by comparing honeys from different sources (Cameroonian and Manuka) and evaluating their functional characteristics. The visual digital and SEM images showed that all three types of honey were successfully loaded within the composite HPMC: gelatin matrix, resulting in clear, transparent and homogenous films that were easy to handle. The presence of honey increased film thickness, % elongation at break, but decreased elastic modulus and tensile strength, suggesting that honey exerted a plasticizing action on the composite polymer matrix. However, all the films demonstrated a good balance between flexibility and toughness, making them suitable for ease of handling and subsequent application onto a wound site. Increasing honey increased mucoadhesion and EWC but caused a general decrease in WVTR with the WVTR values well below that expected for highly exuding wounds. This suggests that while the honey-loaded dressings will remain in place long enough to exert antimicrobial action and maintain a moist environment, the risk of exudate collection is high and therefore more suited for low to medium exuding wounds. Finally, all the honey-loaded dressings, at all concentrations, demonstrated the ability to inhibit the growth of three common wound infection bacteria (*E. coli*, *P. aeruginosa*, and *S. aureus*) and therefore have the potential to be applied to infected chronic wounds. However, the current work involved physico-chemical and in vitro antibacterial testing. Further research will be required using in vitro (fibroblasts and keratinocytes) as well as in vivo wound infection study using an appropriate animal model to confirm their ability to treat infected chronic wounds and enhance wound healing. In addition, the purchasing of the honeys from local market or supermarket presents the challenge of variations between batches which could affect the quality and efficacy of the films, and these will need to be standardized in a future study, including a palynological analysis of honey samples in order to verify and certify their botanical origin.

## 4. Materials and Methods

### 4.1. Materials

HPMC, barium chloride, calcium chloride, sodium chloride, sulfuric acid, bovine serum albumin, were all purchased from Fisher Scientific (Loughborough, UK). Gelatin was obtained from Sigma-Aldrich (Gillingham, UK); Mueller-Hinton broth and agar were obtained from Oxoid Ltd., (Hampshire, UK). Manuka Honey (Pure Gold Active Honey New Zealand) was obtained from a local Holland and Barret in Kent, UK. Pure Cameroonian wild and Cameroonian standard honeys were obtained from local market and supermarket, respectively, in Yaoundé, Cameroon.

### 4.2. Formulation Method Development

#### 4.2.1. Preparation of Blank Films

Aqueous blank gels were prepared at concentrations of 1–2% *w*/*v* comprising HPMC and gelatin in various ratios. Table 1 displays the different polymer ratios and gel concentrations used for preparing blank films. To prepare the gels, each polymer was carefully weighed and dissolved in deionized water on a magnetic stirrer set at 250 rpm at 60 °C until a clear, homogeneous mixture was obtained. Gelatin was added first as it dissolved at 60 °C, and HPMC was gradually added to ensure no clumps developed. The resulting composite gels were left idle for roughly 30 min to settle, and to eliminate any entrapped air bubbles. The solutions were then poured into labelled Petri dishes (diameter = 86 mm) and left to dry in a fan oven at 55 °C for 17–18 h. Upon removal, the films were carefully peeled and stored in a desiccator until required.

#### 4.2.2. Preparation of Honey Loaded Hydrocolloid Films

As noted above, blank films were prepared from 1 to 2% *w*/*v* gels; however, the addition of honey did not mix well with gels containing higher ratios of gelatin as both components created a very viscous and non-homogenously dispersed gel, especially for the 2% films. Therefore, further development for honey-loaded films involved only 1% *w*/*v* gels, containing a lower gelatin ratio, to aid in the effective mixing with honey. The 1% *w*/*v* gels comprising 3:1 ratio of HPMC: gelatin were deemed optimum as they produced films with ideal physical properties, including handling for drug loading. The compositions of gels for film casting are presented in Table 2, showing loading with different concentrations of the three types of honey. The relevant weight of honey was dissolved in 10 mL of water and gently stirred until a clear solution was achieved. The appropriate amounts of polymer were then added together to the remaining volume of water and prepared using the same procedure as the blank films described above. Once prepared, each film was carefully peeled off the Petri dish and placed in individual, tightly sealed plastic bags until needed for further testing.

### 4.3. Physical and Mechanical Characterization

Each film formulated was characterized by evaluating functional properties indicative of effectiveness as a wound dressing.

#### 4.3.1. Film Thickness and Folding Endurance

A scalpel was used to cut the films into 3 × 2 cm^2^ rectangular strips for the evaluation of film thickness. A digital micrometer was used to measure each film at five different locations (four corners and center), and the calculated mean value of the five measurements was recorded as the film thickness. This test was carried out in triplicates for each film. Folding endurance was determined to provide an indication of the flexibility of the film and to assess if the film provided a comfortable and easy application to the wound. The films were cut into 3 × 2 cm^2^ rectangular strips and repeatedly folded at the same place until they broke. The number of times the film could be folded before breaking gave the value of folding endurance. This test was carried out in triplicates for each formulation.

#### 4.3.2. Mechanical (Tensile) Properties

Mechanical characterization tests were conducted using a texture analyzer (HD plus Stable Micro Systems, Godalming, UK) in tensile mode to measure the tensile strength and elasticity of the films. Prior to analysis, the films were cut into dumb-bell-shaped strips to give a gauge length of 30 mm by using a standard dumb-bell shaped cutter press. The cut film strips were clamped into position between the tensile grips of the texture analyzer equipped with a 5 kg load cell, at pretest, test and post-test speeds of 1 mm/s, 2 mm/s and 10 mm/s, respectively, and trigger force of 0.05 N using the software program Texture Exponent 32^®^ (Stable Micro Systems, Godalming, UK). This test was also carried out in triplicates. Using the thickness of the film calculated earlier, the tensile strength (N/mm^2^), elongation at break (%, and elastic modulus were calculated based on Equations (1)–(3):

(1) $$Tensile\;strength=\frac{Force\;at\;break}{cross\;sectional\;area\;of\;the\;film}$$

(2) $$Percentage\;of\;elongation\;at\;break=\frac{increase\;in\;length\;at\;break}{inital\;film\;length}\;\times \;100$$

(3) $$Elastic\;modulus=\frac{slope\;of\;stress\;and\;strain\;curve}{film\;thickness\times cross\;head\;speed}$$

#### 4.3.3. In Vitro Wound Adhesion

Adhesion testing on the films was carried out using the texture analyzer (HD plus Stable Micro Systems, Goldaming, UK) to evaluate the maximum force required to separate the film from the surface of a gelatin mucosal substrate, simulating a wound surface. This in vitro study involved the preparation of the mucosal gelatin substrate, where 6.67 g of gelatin was weighed accurately and mechanically stirred in deionized water (100 mL) at 60 °C until complete dissolution. 20–25 mL of the warm gelatin solution was poured into Petri dishes and placed in a fridge to solidify. To mimic a wound environment further, 0.5 mL of simulated wound fluid (SWF) containing 2% BSA, 0.02 M calcium chloride, 0.4 M sodium chloride, 0.08 M tris(hydroxyl) aminomethane was dissolved in deionized water at a pH of 7.5 and was equilibrated over the surface of the solidified gelatin. Films were cut to the dimensions of the probe (35 mm diameter), and were mounted onto the probe using double-sided adhesive tape and brought into contact with the gelatin surface with an applied force of 0.98 N for 60 s. The texture analyzer was operated using the following settings: pretest, test and post-test speeds of 0.5 mm/s, 0.5 mm/s and 1.0 mm/s, respectively, and trigger force of 0.5 N and return distance of 10 mm. The Texture Exponent 32^®^ software program (Stable Micro Systems, Godalming, UK) was used to determine the PAF, cohesiveness, and WOA for each sample. This was repeated 3 times, and the mean was calculated.

### 4.4. Water Handling Properties

The water handling properties were evaluated by measuring the equilibrium water content (EWC) and water vapor transmission rate (WVTR).

Equilibrium water content (EWC) tests were performed to investigate the maximum water holding capacities of the film dressings in simulated wound fluid (SWF) at a pH of 7.5. The films were cut into square strips, accurately weighed and placed into 10 mL of SWF over 24 h in an incubator oven set to 37 °C. The films were removed from the oven after 24 h, wiped gently with paper towels to remove excess SWF and weighed again. Each measurement was performed in triplicate (*n* = 3). The *EWC* (%) (*n* = 3) was calculated using Equation (4) below.

(4) $$EWC\left(\%\right)=\frac{Ws-Wi}{Ws}\times \;100$$

where *Ws* is the swollen weight and *Wi* is the initial weight before immersion into SWF.

To determine the WVTR, 8 mL of deionized water was measured and poured in a Falcon tube (16.66 mm diameter). Strong adhesive glue was placed on the rim of Falcon tube and a film cut into 3 × 3 mm^2^ square strip was placed on top to seal it. This process was repeated for each formulation in triplicates. The initial weight of the Falcon tube and film was recorded and then placed in an incubator at 35° C. The incubator with an air flow vent was used to ensure continuous air flow in the samples. Samples were weighed again after different time intervals (every hour for 6 h, and then 24 h). The WVTR was calculated using Equation (5) below.

(5) $$WVTR=\frac{Wi-Wt}{A}\times 10^{6}/{g\;m}^{2}\;{\mathrm{day}}^{-1}$$

where *Wt* is the swollen weight at 24 h, *Wi* is the initial weight before incubation in SWF and *A* is the surface area of sphere used for the testing.

### 4.5. Analytical Characterization

#### 4.5.1. Scanning Electron Microscopy (SEM)

SEM was used to examine the surface morphology of the films using a Hitachi triple detector scanning electron microscope (Hitachi SU8000, Hitachi-high technologies, Krefeld, Germany). Each sample was cut into 3 × 3 mm^2^ strips and was mounted onto 12 mm aluminium stubs with double-sided carbon tape. Prior to analysis, films were coated with chrome under vacuum to make them electrically conductive. The images were acquired at 400 and 1.5 k magnifications at an accelerating voltage of 2 kV.

#### 4.5.2. Fourier Transform Infrared Spectroscopy (FTIR)

A Nicolet ATR-FTIR spectrophotometer (Thermo Scientific, Waltham, MA, USA) was used to characterize the uniformity of the films. To do this, the FTIR was connected with a SMART arc to determine the polymer and drug interactions in the hydrocolloid films. A small piece of film (roughly 1 × 1 cm^2^) was cut and placed on the surface of the ATR crystal. Force was applied by a pressure clamp to ensure direct contact between the sample and crystal. The spectra were recorded at a resolution of 4 cm^−1^ and at a wavelength between 650 and 4000 cm^−1^. This process was repeated for all samples including the starting materials (polymers and different honeys).

#### 4.5.3. X-Ray Diffraction (XRD)

XRD was used to analyze the physical form of the films by using a D8 Advanced X-ray Diffractometer (Bruker, Coventry, UK) in 2θ transmission mode. Prior to analysis, films were cut and folded to fit into the sample cell. The diffractometer was equipped with Cu Kα radiation at 40 kV and 40 mA and a primary Göbel mirror was used as an X-ray beam in a parallel sequence to remove CuKβ radiation. Furthermore, a 0.6 mm Soller slit and secondary Soller slit at 2.5° were used with a sample rotation of 30 rpm. A LynxEye silicon strip was used as a position sensitive detector and a set opening of 3°, with the LynxIris at a setting of 6.5 mm. The data was collected at a range of 3–40° 2θ (two-theta) with a step size of 0.03° and set at a counting time of 0.4 s per step. In total, there were 176 active channels present in the detector, with each sample scanned for approximately 5 min. The software used to collect data was DIFFRAC plus XRD Commander version 2.6.1 Bruker-AXS (Bruker, Coventry, UK).

### 4.6. Antimicrobial Susceptibility Testing

The antimicrobial action was conducted by following the Kirby and Bauer standardized method for three common wound infection causative bacteria *Escherichia coli*, *Pseudomonas aeruginosa*, and *Staphylococcus aureus*. Mueller−Hinton (38 g) agar was suspended in 1 L of boiled deionized water until complete dissolution. After transferring medium to a 1 L glass bottle, the medium was autoclaved at 121 °C for 1 h. A Bunsen burner was used during all processes to avoid contamination. Upon cooling, 20–25 mL of the medium was plated onto sterile Petri dishes, inverted once cooled, and stored in the fridge to solidify.

#### 4.6.1. McFarland Standard

McFarland standard was used to allow visual comparison of the bacterial density of the inoculated bacteria prepared earlier. To prepare 0.5 M of McFarland standard, 0.5 mL aliquot of a 0.048 mol/L BaCl_2_ (1.175% *w*/*v* BaCl_2_2H_2_O) to 99.5 mL of 0.18 mol/L H_2_SO_4_ (1% *v*/*v*) was suspended and stirred until uniformly dispersed. The McFarland standard was stored in the dark at room temperature until required.

#### 4.6.2. Preparation of Inoculum

A sterile inoculating loop was used to isolate 3 to 4 colonies of the organisms (*E. coli*, *P. aeruginosa*, and *S. aureus*) to be tested, suspended in 3 mL of MH medium and the resulting suspension was vortexed and incubated for 3 h. The turbidity of the bacteria was then adjusted by adding drops of Mueller−Hinton medium until the turbidity resembled the 0.5 M McFarland standard with the help of a Wickerham card. Each prepared inoculum was used within 30 min of preparation.

#### 4.6.3. Inoculation of MH Plate for Disc Diffusion Susceptibility Test

As the tested organisms must be in the log phase of growth to achieve viable results, the correct volume of adjusted bacteria needed was calculated. The calculated aliquot (300 µL) of the adjusted bacterial inoculum and 2.97 mL of Mueller−Hinton medium (3 mL in total) was suspended in an Eppendorf tube and vortexed. Mueller−Hinton agar plates were labelled accordingly, and a sterile swab was taken and dipped into the appropriate Eppendorf tube and pressed against the tube to remove excess fluid. The Mueller−Hinton agar plate was streaked with the swab in a ‘zigzag’ motion over the surface of the plate, turned clockwise and streaked again, a total of 3 times.

After swabbing, plates were left to dry for 10 min leaving the lid slightly ajar. Pre-cut circular film discs (15 mm diameter) were centrally placed on the surface of the agar carefully using sterile forceps, left for another 10 min, and the plates were inverted and placed in the incubator at 35 °C for 24 h. Following incubation, the zone of inhibition was measured using a ruler and tested in triplicates for each organism.

#### 4.6.4. Broth Dilution Test

The broth dilution test was used to determine the minimum inhibitory concentration (MIC) of the samples. Thirteen 50 mL Falcon tubes (1 for blank film and 12 honey loaded film containing the three types of honeys at different concentrations) for *E. coli*, *P. aeruginosa*, and *S. aureus*, were assembled, and labelled accordingly. Aliquots (100 μL) of the adjusted inoculum were then carefully added to each centrifuge tube and 9.9 mL of Mueller−Hinton medium was then added (10 mL in total), after which a pre-cut (15 mm diameter) film was added to the respective tube. The Falcon tubes were then placed in a shaking incubator set at 180 RPM at 37 °C for 24 h. Following incubation, the tubes were removed and arranged to determine MIC as well. Further, the Falcon tubes were also used to determine the minimum bactericidal concentration (MBC) and cell count. For this, Mueller−Hinton agar plates were labelled appropriately, with 100 μL of the solution from the Falcon tubes added to its respective plate. A spreader was used to spread it thoroughly and incubated for 24 h once completed. This entire process was repeated for accuracy for each bacterium.

### 4.7. Statistical Analysis

The relevant quantitative (thickness, mechanical strength, mucoadhesion, moisture handling and ZOI) data were subjected to statistical analysis using simple *t*-test and/or one way ANOVA followed by Bonferroni post hoc *t*-test with the help of Microsoft Excel to compare between the blank and honey-loaded films as well as between the different honey-loaded (different sources and concentrations) films. Significant difference was set at *p*-values < 0.05 or *p*-values < 0.01.

## Figures and Tables

**Table 1:** Composition of preliminary blank films prepared from 1 to 2% *w*/*v* composite aqueous gels.

Formulation	HPMC: Gelatin	HPMC (mg)	Gelatin (mg)	Water (mL)
	1% Film			
BLA1	1:1	250	250	50
BLA2	3:1	375	125	50
BLA3	1:3	125	375	50
	**2% film**			
BLA4	1:1	500	500	50
BLA5	3:1	750	250	50
BLA6	1:3	250	750	50

**Table 2:** Composition of the honey loaded composite HPMC and gelatin film dressings containing different amounts of honey.

Formulation	HPMC: Gelatin Ratio	HPMC (mg)	Gelatin (mg)	Honey (mg)	Water (mL)
CWH1	3:1	250	750	75	50
CWH2				125	
CWH3				250	
CWH4				375	
CSH5	3:1	250	750	75	50
CSH6				125	
CSH7				250	
CSH8				375	
MH9	3:1	250	750	75	50
MH10				125	
MH11				250	
MH12				375	

Abbreviations: CWH—Cameroonian wild honey, CSH—Cameroonian standard honey, MH—Manuka honey.

**Table 3:** Equilibrium water content (EWC) and water vapor transmission rate (WVTR) for the different honey–loaded films compared to the optimum blank films. Significant difference at * *p* < 0.05; ** *p* < 0.01; ns (nonsignificant) (compared to BLA2).

Formulation	EWC (%) (±SD)	WVTR (gm^2^ Day^−1^) (±SD)
CWH1	47.5 ± 2.3 *	900 ± 212 **
CWH2	63.4 ± 1.5 *	759 ± 117 **
CWH3	65.0 ± 5.9 *	879 ± 527 **
CWH4	72.0 ± 3.0 *	759 ± 27 **
CSH5	31.7 ± 1.6 ns	754 ± 118 **
CSH6	45.6 ± 3.0 *	405 ± 127 *
CSH7	63.8 ± 2.6 *	568 ± 150 *
CSH8	64.1 ± 1.8 *	576 ± 102 *
MH9	32.9 ± 1.6 ns	536 ± 87 **
MH10	53.7 ± 1.9	352 ± 67 ns
MH11	63.5 ± 1.2 *	298 ± 37 ns
MH12	68.1 ± 2.0 *	479 ± 95 *
BLA2	38.3 ± 1.8	320 ± 60

**Table 4:** ZOI of final films for *E. coli*, *S. aureus*, and *P. aeruginosa.* (mean ± SD).

Formulation	ZOI ± SD (mm)		
	*S. aureus*	*P. aeruginosa*	*E. coli*
BLA2	0.0	0.0	0.0
CWH1	3.1 ± 0.1	2.5 ± 0.2	2.1 ± 0.1
CWH2	3.6 ± 0.1	3.0 ± 0.2	2.8 ± 0.1
CWH3	5.8 ± 0.2	5.5 ± 0.1	5.5 ± 0.2
CWH4	6.5 ± 0.1	6.0 ± 0.2	5.5 ± 0.2
CSH5	2.1 ± 0.2	2.0 ± 0.3	2.0 ± 0.1
CSH6	3.3 ± 0.1	2.9 ± 0.1	2.5 ± 0.1
CSH7	4.5 ± 0.1	3.8 ± 0.1	3.5 ± 0.2
CSH8	6.1 ± 0.2	5.5 ± 0.1	5.5 ± 0.1
MH9	2.4 ± 0.2	2.2 ± 0.2	2.1 ± 0.2
MH10	3.4 ± 0.1	3.2 ± 0.3	3.1 ± 0.1
MH11	5.0 ± 0.1	4.5 ± 0.2	4.3 ± 0.1
MH12	6.0 ± 0.1	5.7 ± 0.1	5.5 ± 0.1

## Data Availability

The original contributions presented in this study are included in the article/Supplementary Materials. Further inquiries can be directed to the corresponding author.

## Supplementary Materials

The following supporting information can be downloaded at: https://www.mdpi.com/article/10.3390/gels11070557/s1, Figure S1: Images of blank films containing HPMC and gelatine: BLA1 (left), BLA2 (middle) and BLA3 (right), Figure S2: HPMC only films prepared from (a) 1% (showed a light yellow tint, did not retain shape, sticky) and (b) 2% (slight yellow tint, did not retain shape sticky, did not dry thoroughly) gels, loaded with 5% *w*/*v* honey, Figure S3: Formulation development and optimization of composite HPMC: gelatin (H:G) films prepared from gels 1 and 2% w/w gels containing 5% *w*/*v* honey, Figure S4: ZOI for CWH3 (top) and CWH4 (bottom) for *E. coli*, *S. aureus*, and *P. aeruginosa*, Figure S5: ZOI for CSH7 (top) and CSH8 (bottom) for *E. coli*, *S. aureus*, and *P. aeruginosa*, Figure S6: ZOI for MH11 (top) and MH12 (bottom) for *E. coli*, *S. aureus*, and *P. aeruginosa*, Figure S7: MIC determination of the honey-loaded films against the infection causative organisms, Table S1: Summary of the wound healing effects of honey.
