# Supplementary material for: Composite HPMC-Gelatin Films Loaded with Cameroonian and Manuka Honeys Show Antibacterial and Functional Wound Dressing Properties
Source: Gels. 2025 Jul 19;11(7):557. doi: 10.3390/gels11070557 (PMC12294384; doi:10.3390/gels11070557)
Supplement: Supplementary file 1 [file gels-11-00557-s001.zip › gels-3732742-supplementary.pdf]

Table S1. Summary of the wound healing effects of honey (4).

| General effects of honey on wound healing | Suggested rationale |
|---|---|
| Accelerated wound healing | Stimulation of cytokine production |
| Antiseptic and analgesic properties | Analgesic effects of honey and antiseptic properties found to be effective against a range of microbes |
| Control of bacterial contamination | Viscosity of honey provides a protective barrier |
| Reduced inflammation, reduced pain | Number of inflammatory cells reduced |
| Promotes epithelialization of damaged tissue | Analgesic effects of honey |
| Facilitates autolytic debridement | Facilitates the autolytic action of proteases |
| Inhibits adhesion of bacteria in wound | Honey possesses anti-adhesive properties against wound pathogens |
| Promotes moist wound healing | Osmolarity extracts fluid from underlying tissue |
| Reduces malodor | Bacterial preference of sugar instead of protein reduces malodorous compounds |
| Honey does not adhere to wound surface | Viscosity of honey provides interface between wound bed and dressing |
| Regulates oxidative stress | Inhibition of reactive oxygen species |

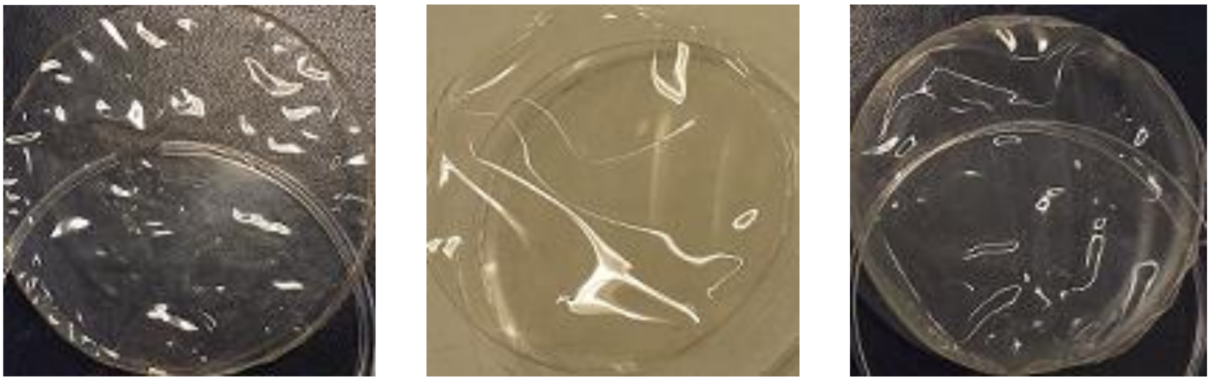

**BLA1** **BLA2** **BLA3**

Figure S1: Images of blank films containing HPMC and gelatine: BLA1 (left), BLA2 (middle) and BLA3 (right).

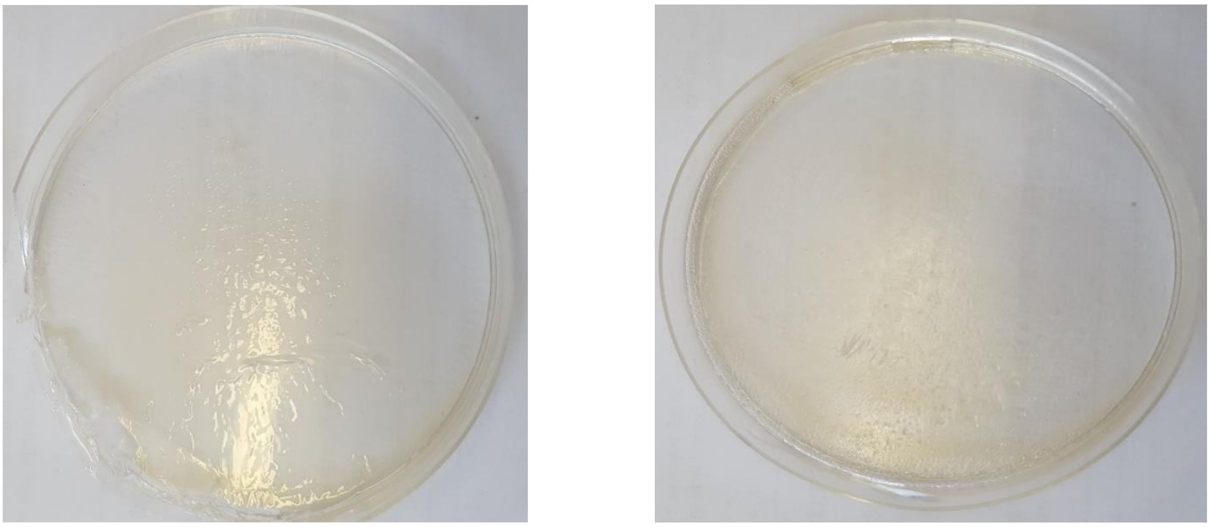

**1% w/v** **2% w/v**

Figure S2 HPMC only films prepared from (a) 1% (showed a light-yellow tint, did not retain shape, sticky ) and (b) 2% (slight yellow tint, did not retain shape sticky, did not dry thoroughly) gels, loaded with 5% w/v honey

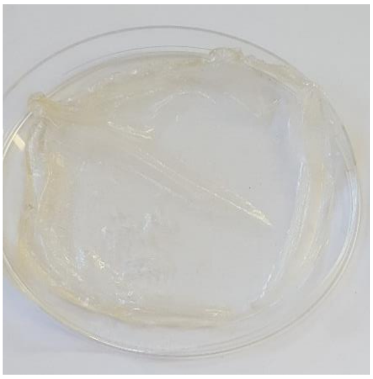

**1% w/v H:G 1:1**

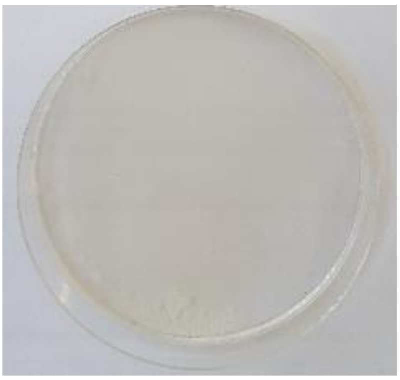

**1% w/v H:G 3:1**

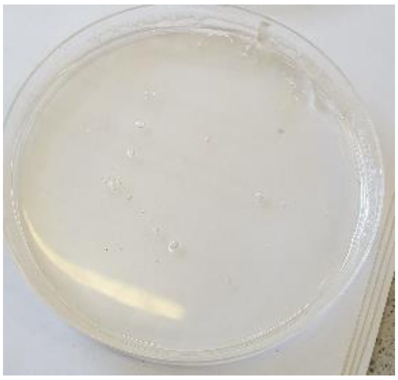

**1% w/v H:G 1:3**

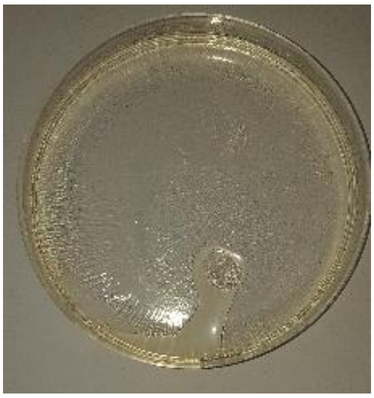

**2% w/v H:G 1:1**

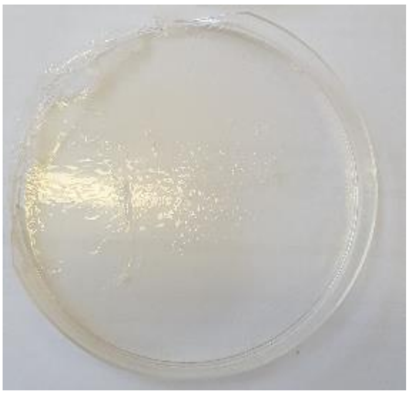

**2% w/v H:G 3:1**

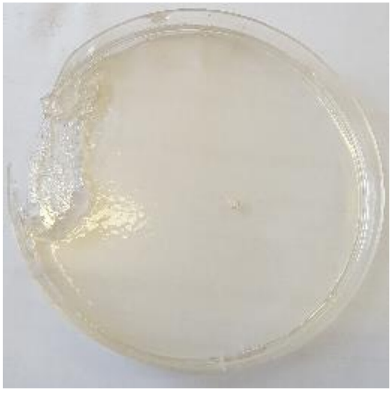

**2% w/v H:G 1:3**

Figure S3 Formulation development and optimization of composite HPMC: gelatin (H:G) films prepared from gels 1 and 2% w/w gels containing 5% w/v honey

1% (H:G) 1:1 honey-loaded films were clear, sticky, homogenously dispersed, did not retain shape.
1% (H:G) 3:1 honey-loaded films were clear, flexible, homogenously dispersed and retained shape.
1% (H:G) 1:3 honey-loaded films were clear, solvent did not evaporate fully, showed large air bubbles that could not be released, did not retain shape.

2% (H:G) 1:1 honey-loaded films were clear, solvent did not evaporate fully, homogenously dispersed but did not retain shape.
2% (H:G) 3:1 honey-loaded films were clear, sticky, homogenously dispersed and retained shape
2% (H:G) 1:3 honey-loaded films were clear, sticky and showed incomplete solvent evaporation

**CWH3**

**CWH4**

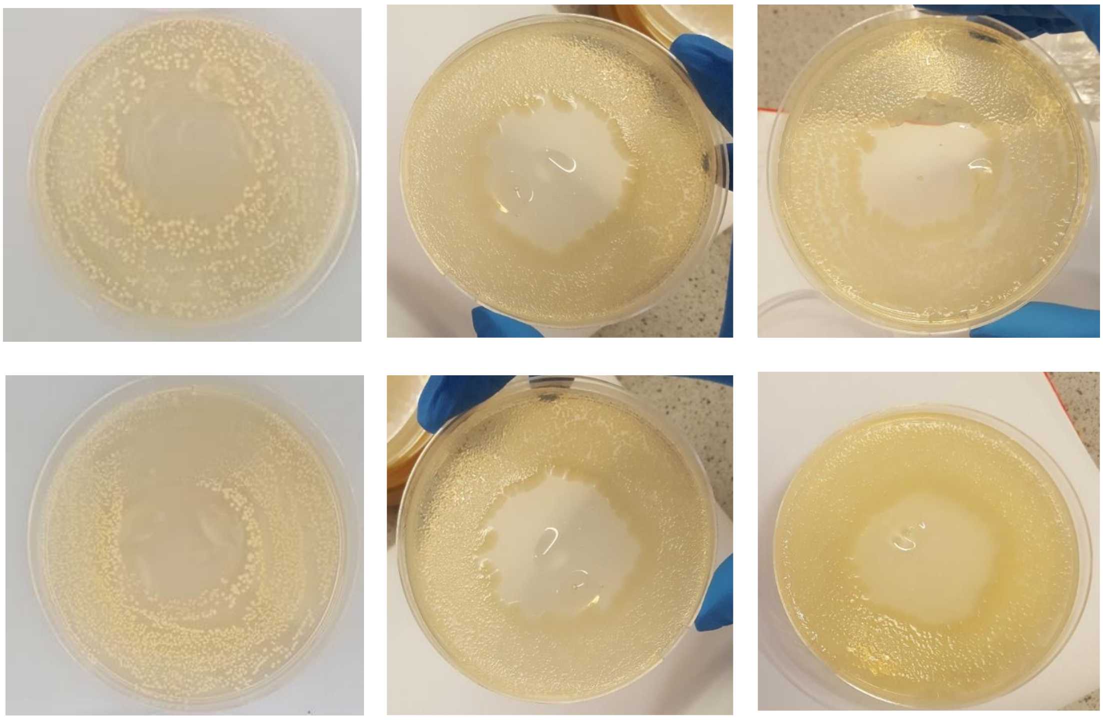

**E. coli** **S. aureus** **P. aeruginosa**

Figure S4: ZOI for CWH3 (top) and CWH4 (bottom) for *E. coli, S. aureus,* and *P. aeruginosa*

CSH7

CSH8

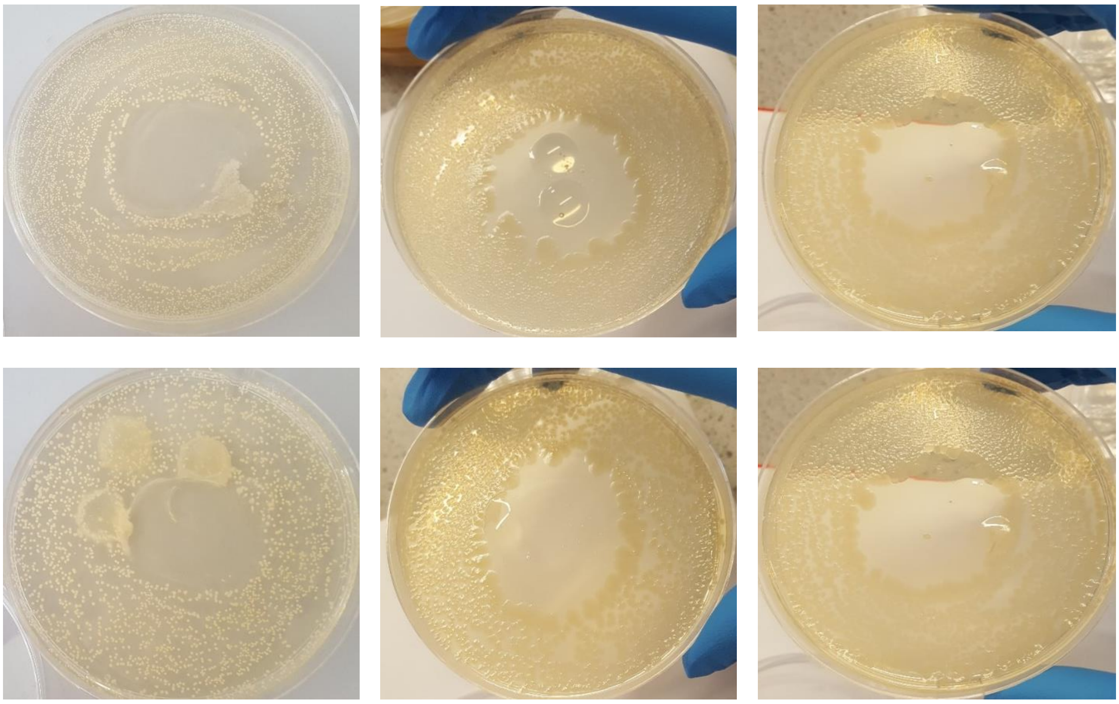

**E. coli** **S. aureus** **P. aeruginosa**

Figure S5: ZOI for CSH7 (top) and CSH8 (bottom) for *E. coli, S. aureus,* and *P. aeruginosa*

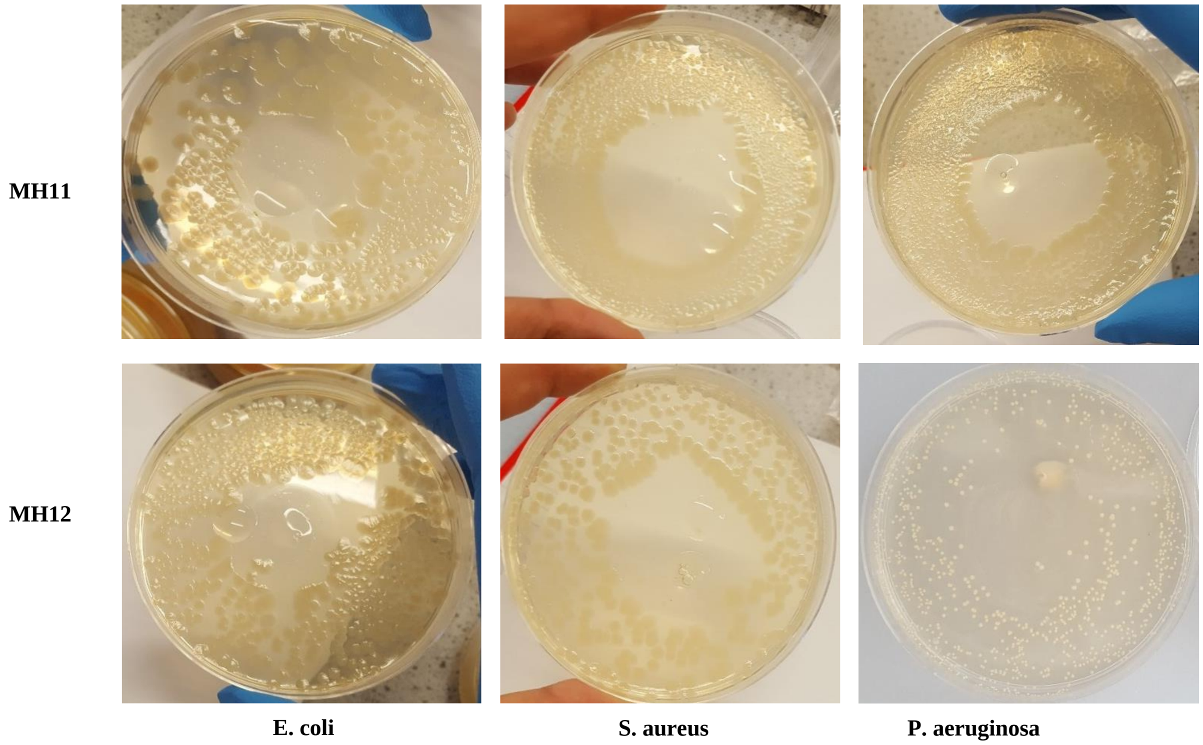


Figure S6: ZOI for MH11 (top) and MH12 (bottom) for *E. coli, S. aureus,* and *P. aeruginosa.*

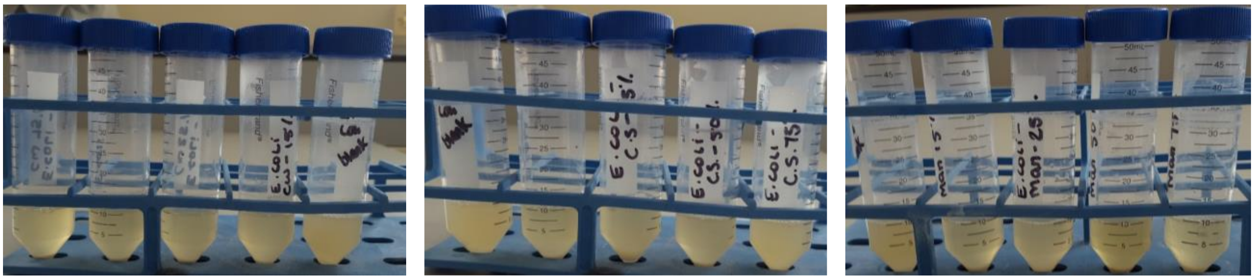

**CWH** **CSH** **MH**

**E coli**

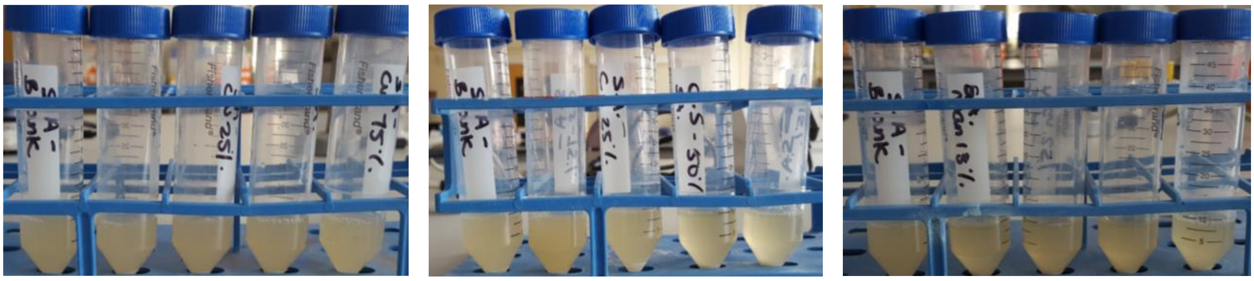

**CWH** **CSH** **MH**

**Staph aureus**

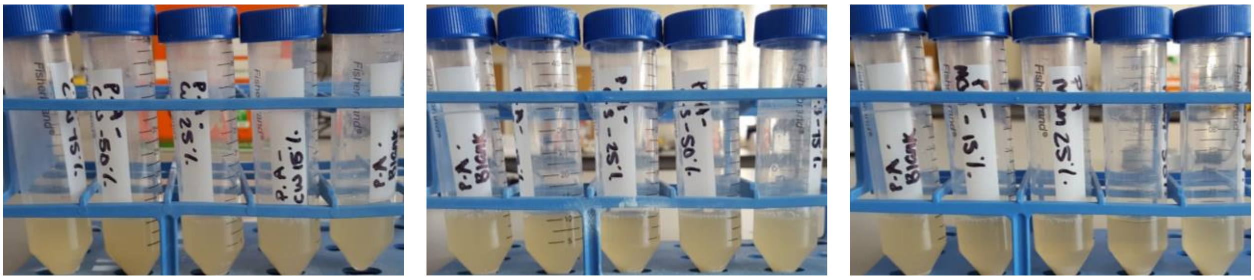

**CWH** **CSH** **MH**

**Ps aeruginosa**

Figure S7: MIC determination of the honey-loaded films against the infection causative organisms.
